# Low-to-Mid-Frequency Monopole Source Levels of Underwater Noise from Small Recreational Vessels in the St. Lawrence Estuary Beluga Critical Habitat

**DOI:** 10.3390/s23031674

**Published:** 2023-02-03

**Authors:** Dominic Lagrois, Camille Kowalski, Jean-François Sénécal, Cristiane C. A. Martins, Clément Chion

**Affiliations:** 1 Département des Sciences Naturelles, Université du Québec en Outaouais, Ripon, QC J0V 1V0, Canada; camille.kowalski06@gmail.com (C.K.); senecal.jean-francois.3@courrier.uqam.ca (J.-F.S.); clement.chion@uqo.ca (C.C.); 2 Parc Marin du Saguenay-Saint-Laurent, Tadoussac, QC G0T 2A0, Canada; cristiane.albuquerque@pc.gc.ca

**Keywords:** underwater acoustics, vessel underwater noise determination, anthrophony, noise pollution, hydrophone-based observations, species at risk, passive acoustic monitoring

## Abstract

Anthropogenic noise from navigation is a major contributor to the disturbance of the acoustic soundscape in underwater environments containing noise-sensitive life forms. While previous studies mostly developed protocols for the empirical determination of noise source levels associated with the world’s commercial fleet, this work explores the radiated noise emitted by small recreational vessels that thrive in many coastal waters, such as in the St. Lawrence Estuary beluga population’s summer habitat. Hydrophone-based measurements in the Saguenay River (QC, Canada) were carried out during the summers of 2021 and 2022. Shore-based observations identified 45 isolated transits of small, motorized vessels and were able to track their displacement during their passage near the hydrophone. Received noise levels at the hydrophone typically fell below the hearing audiogram of the endangered St. Lawrence Estuary beluga. Monopole source levels at low frequencies (0.1–≲2 kHz) held on average twice the acoustic power compared to their mid-frequency (≳2–30 kHz) counterparts. The speed over ground of recreational vessel showed a positive correlation with the back-propagated monopole source levels. Estimations of the mid-frequency noise levels based on low-frequency measurements should be used moderately.

## 1. Introduction

Vessels’ radiated noise contributes to the increase of ambient noise levels in coastal shallow waters [1,2]. For species with frequency-specific hearing sensitivity, interactions with marine traffic can lead to behavioural modifications [3], variations in frequency and amplitude of the vocalization regime [4], sound masking that could shorten distances for efficient communication between individuals [5,6], and temporary-to-permanent hearing damage [7,8].

The deployment of hydrophonic arrays can be used to quantify levels of noise radiated at the source of nearby transiting ships [9,10]. Information gathered from recording missions can then be used to develop numerical/empirical models of the radiated source levels [11,12,13,14] which, in turn, are used to supply frequency-dependent source characterization in propagation loss models (e.g., [15,16]).

Previous efforts in that matter have mainly focused on post-WWII military warcrafts [17,18] and merchant vessels [19,20,21], while the acoustic properties and repercussions of small vessels [22,23] have been less explored in the literature. This work extends the current knowledge on ships as acoustic sources to the class of small motorized recreational vessels, which account for a large fraction of the maritime traffic during the high season in the vicinity of touristic harbours [24].

The main objectives of this study were to:

1. Compute monopole source levels from low-to-mid frequencies of motorized recreational vessels in isolated transits observed during the summers of 2021 and 2022 in the Saguenay River (QC, Canada);
2. Investigate the correlations between monopole source levels and small vessels’ static (e.g., subtype/category) and dynamic (e.g., speed) parameters;
3. Discuss the potential effect of these recreational vessels on a portion of the summer habitat of the St. Lawrence Estuary beluga, an endangered species protected under the Canada’s Species at Risk Act [25].

## 2. Material

### 2.1. Shore-Based Observations

All large commercial ships are equipped with GPS tracking systems and VHF communication ensuring a constant supply of information such as unique identification, status, position, course, and speed to the automatic identification system (AIS). The AIS database allows one to track a given ship’s position with respect to a deployed hydrophone in order to correctly interpret the sound pressure levels received during its transit. Small recreational vessels are not required to be equipped with AIS transponders. Hence, retrieving their position, course, and speed with respect to a deployed hydrophone becomes a challenge. Shore-based theodolite measurements [26] were used in this project to fill this gap.

**Figure 1:**
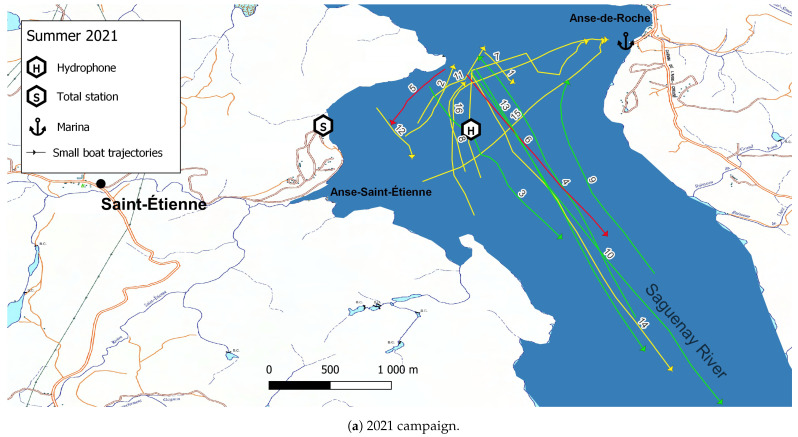

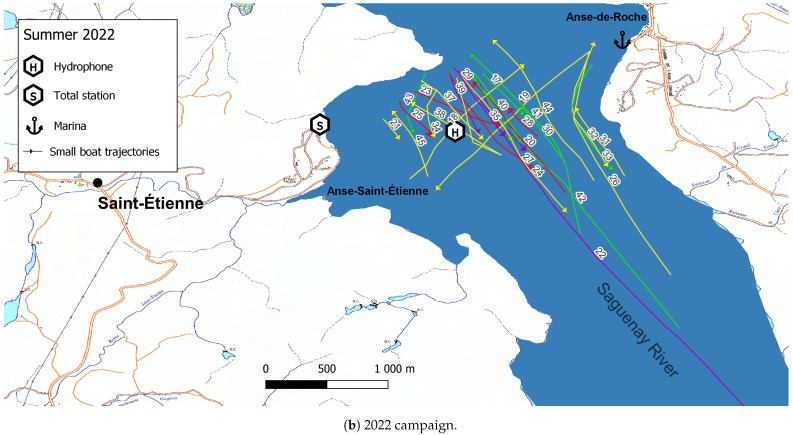
Zone of interest along the Saguenay River. Positions of the shore-based Leica TS06 total station, hydrophone, and marina at Anse-de-Roche are identified. Trajectories followed by 45 isolated recreational vessels during the summers of (**a**) 2021 and (**b**) 2022 are shown. Trajectories are colour-coded according to the vessel’s type: red for Zodiac^TM^, yellow for speedboats, green for cruisers, and purple for sailboats. Labels refer to individual entry lines in Table 1.

A Leica TS06 total station was set up at $\phi_{\mathrm{TS}06}$ = 48.2092094°, $\lambda_{\mathrm{TS}06}$ = −69.9081906° on a cliff of height $h_{c}$ = 50.51 m above sea level (see Figure 1). The total station provided horizontal and vertical angles of pointed targets with respect to zero-references [27]. The precision on the angle measurements was of 5″.

### 2.2. Acoustic Observations

Autonomous hydrophone-based measurements were carried out in 2021 from July 7th to September 17th and in 2022 from July 15th to September 8th. A ST300 HF hydrophone (SoundTrap Ocean Instruments, New Zealand) was deployed at $\phi_{2021}$ = 48.2087833°, $\lambda_{2021}$ = −69.8919500° and $\phi_{2022}$ = 48.2093167°, $\lambda_{2022}$ = −69.8934167°, roughly 1 km from the shore at the junction between Anse-Saint-Étienne and the Saguenay River (see Figure 1).

Each summer, the hydrophone was attached to a weighted tripod which was lowered on the seabed. Once deployed, the hydrophone’s resting depth was about 1 m above from the sediments’ floor. The Canadian Hydrographic Service gives a water column height $z_{0}$ of 97 and 93 m, respectively, at the deployment position of 2021 and 2022. The hydrophone’s sampling rate was 192 kHz and the end-to-end system sensitivity was −175.7 dB re 1 VμPa^−1^ [28].

## 3. Methods

### 3.1. Shore-Based Measurements

On the opposite shore of the Saguenay River, a landmark located near the marina of Anse-de-Roche, right in front of the land-based station (see Figure 1), was designated as the horizontal zero-reference from which the horizontal angles ( $\theta_{h}$ ) of pointed targets would be measured. The difference between the horizontal zero-reference and the true north was ΔΘ = 67.77778°, measured clockwise. No vertical zero-reference was required as, by default, 0° is the horizon and 180° is the opposite direction. The vertical angles ( $\theta_{v}$ ) of pointed targets were measured downward towards the bay from the observer’s point of view on the cliff.

The ground distance *d* between the observer and a given vessel *i* was obtained by:

(1) $$d_{i}\;=\;\frac{h_{i,eff}}{\tan(\theta_{i,v})},$$

where the effective height $h_{i,eff}$ of the total station with respect to target *i* was provided by:

(2) $$h_{i,eff}\;=\;h_{c}\;+\;h_{obs}\;-\;h_{i,t},$$

where $h_{c}$ is the cliff’s height (see Section 2.1), $h_{obs}$ is the observer’s eye height, and $h_{i,t}$ was the tides’ height in Anse-Saint-Étienne at the time of the observation of vessel *i*.

We followed the formalism developed by [29] to estimate the target’s position using the observer’s position on the cliff, the observer-to-vessel ground distance, and the vessel’s true course. First, the latitude $\phi_{i}$ of the pointed target *i* was provided by:

(3) $$\phi_{i}\;=\;\sin^{-1}\left[\sin\left(\phi_{\mathrm{TS}06}\right)\cos\left(\frac{d_{i}}{R_{⊕}}\right)+\cos\left(\phi_{\mathrm{TS}06}\right)\sin\left(\frac{d_{i}}{R_{⊕}}\right)\cos\left({℘}_{i}\right)\right],$$

where the Earth’s radius $R_{⊕}$ = 6.371 × 10^6^ m and the vessel’s true course ${℘}_{i}$ was given by:

(4) $${℘}_{i}\;=\;\theta_{i,h}\;+\;\Delta \Theta .$$

Once the value for $\phi_{i}$ was retrieved from Equation (3), the longitude $\lambda_{i}$ of the pointed target *i* was provided by:

(5) $$\lambda_{i}\;=\;\;\mathrm{mod}\;\left.\left.(\lambda_{\mathrm{TS}06}+\tan^{-1}\left[\frac{\sin\left({℘}_{i}\right)\sin\left(\frac{d_{i}}{R_{⊕}}\right)\cos\left(\phi_{\mathrm{TS}06}\right)}{\cos\left(\frac{d_{i}}{R_{⊕}}\right)-\sin\left(\phi_{\mathrm{TS}06}\right)\sin\left(\phi_{i}\right)}\right],2\pi \right),\right.$$

where the plus (+) sign preceding the tan^−1^ function differed from the minus (−) sign shown in [29]’s equation due to the fact that we were using negative longitudes.

Hence, Equations (3) and (5) could be used to track the position of recreational vessels as long as the observer on the cliff could maintain a visual line-of-sight with the pointed target. Depending on the traffic, horizontal ( $\theta_{h}$ ) and vertical ( $\theta_{v}$ ) angles were recorded approximately every minute. Static (i.e., type of small vessel, size category, whether motors were in-board or out-board, and if motors were out-board, the number, manufacturer, and horsepower) and dynamic characteristics (i.e., orientation, speed category, activity) were also noted. Finally, pictures of the tracked small vessels were taken. Orientations were deduced from successive angle measurements assuming a linear trajectory between each of them.

Shore-based observations did not follow a strict schedule throughout the summers of 2021 and 2022 but rather depended on the meteorological conditions (e.g., no observations were carried out on rainy days) and the on-site availability of the Ph.D. student (C. Kowalski).

Environmental conditions such as visibility, sea state, cloud cover, and water reflections were measured at the beginning of each day and updated if any changes occurred.

### 3.2. Bandwidth of Interest

Appendix A discusses how acoustic data were processed following the hydrophone’s retrieval after each recording campaign. Matlab^®^-supported PAMGuide [30] was used to convert the waveforms of the recorded signal to sound pressure levels (SPLs), hereafter referred to as the frequency-dependent received noise levels (RLs).

RLs spectra were computed from $f_{0}$ = 0.01 kHz to $f_{1}$ = 96 kHz. Shallow-depth environments act as a barrier for the propagation of low-frequency signals. The lower boundary $f_{0}$ is the cut-off frequency expected in a medium of depth ${\bar{z}}_{0}$ , speed of sound $c_{w}$ , and sediment deposits of the inner speed of sound $c_{b}$ :

(6) $$f_{0}\;=\;\frac{c_{w}}{4{\bar{z}}_{0}\sqrt{1-{\left(c_{w}/c_{b}\right)}^{2}}},$$

where ${\bar{z}}_{0}$ = 95 m, $c_{w}$ ∼1440 m s^−1^ (Observatoire global du Saint-Laurent), and $c_{b}$ ∼ 1575 m·s^−1^ [31].

The higher boundary $f_{1}$ was half the hydrophone’s sampling rate, in agreement with the Nyquist theorem [32].

### 3.3. RLs Spectra of Interest

Shore-based observations’ logbooks (Section 3.1) were used to isolate specific moments during the 2021–2022 campaigns when a single motorized recreational vessel was present in the hydrophone’s vicinity without any contamination from other sound sources, i.e., low sea state, no precipitation, no apparent marine mammals, no other nearby vessels. The observations were opportunistic with no control on the vessels’ speed and direction, and no specific information on the engines’ type, RPM, and propellers’ characteristics. Table 1 shows the terrain parameters for 45 events fulfilling these conditions; 7 rigid-hulled inflatable boats (Zodiac^TM^), 21 speedboats, 13 cruisers, and 4 sailboats were identified. For each vessel, the trajectory that was sampled by the total station is shown in Figure 1 with the corresponding label, from 1 to 45, provided in the first column of Table 1. Relatively short trajectories (e.g., events 1 in 2021 and 21 in 2022) were the consequence of potential sources of noise contamination (e.g., another small vessel) that suddenly appeared in the observer’s field-of-view. The table’s right-hand column gave the distances at the closest point of approach (CPA) to the hydrophone. The speed over ground (SOG) was computed assuming a constant speed and a linear displacement between two successive shore-based measurements close to the CPA. Vessels’ positions ( $\phi_{i,\mathrm{CPA}}$ , $\lambda_{i,\mathrm{CPA}}$ ) at the CPA were provided by Equations (3) and (5).

**Figure 2:**
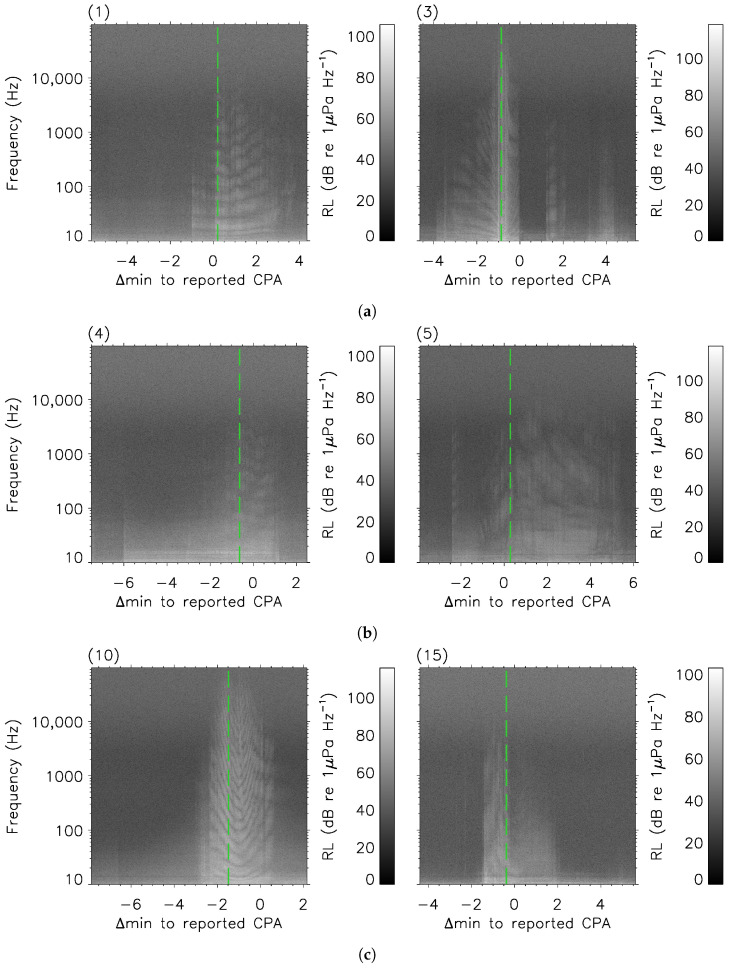
Examples of spectrograms for events of isolated recreational vessels during the 2021–2022 campaigns along the Saguenay River. The long-dashed vertical green line in each panel indicate the time of the highest signal integrated across the frequency domain. Slight offsets (typically <60 s) between these vertical green lines and the reported times for the CPA passages (0 along the abscissa) in Table 1 are noted (see text). Negative (positive) Δmin values indicate that the vessel was approaching (distancing) the hydrophone. Panels’ numbers refer to the vessels’ labels in Table 1. (**a**) Events 1 and 3; (**b**) events 4 and 5; (**c**) events 10 and 15.

Noteworthy examples of spectrograms of vessels’ passage near the hydrophone are shown in Figure 2. Offsets between the time of highest received signal and the time of the reported CPAs reflect either a rate of the shore-based measurements (about 1 min^−1^; see Section 3.1) being too low, the hydrophone’s internal clock drift, or noise reverberations attributed to mechanical vibrations along the vessels’ submerged parts [33]. For each of the 45 events listed in Table 1, the moment at which the received signal was maximal is treated, in the following discussion, as the predicted moment of the CPA passage according to the total station’s data.

Hence, the 45 RLs spectra, identified by the vertical green line for the specific examples of Figure 2, were said to be the spectra of interest (i.e., at the CPA). The methods for the broadband calculations are described in Appendix B.

The observer operating the total station noticed the presence of belugas in or close to Anse-Saint-Étienne swimming north up the Saguenay River during events (5), (6), and (24). These events are starred in Table 1. A visual investigation of the corresponding spectrogram for these events and a careful listening of the audio WAV files (see Appendix A) revealed no indication of vocalizations recorded at the hydrophone’s positions.

### 3.4. Backpropagation

The backpropagation from the receiver to the targets’ position was estimated using the split-step Padé approximation of the parabolic equation method [34] in the low-frequency domain (*f* < *f*_0.5_) and the ray-tracing approach for sound beams [35] in the mid-frequency domain (*f* ≥ *f*_0.5_). The RAM and Bellhop models were used to quantify the frequency-dependent transmission loss (TL_RAM_, TL_Bellhop_) that resulted from the geometric dilution of the sound signal as it travelled through water.

The use of numerical algorithms for transmission loss are here highly recommended by comparison to the standard geometrical spreading. The klog(r) -law could indeed be reliable at sub-km ranges although quasi-semi-infinite (or very deep) environments are required. Lloyd’s mirror (surface) effects and secondary reflections on the sea floor make spreading laws inaccurate in shallow-to-intermediate waters (see Figure 1 of [36]). On the other hand, numerical algorithms imply computing times that increase nonlinearly with the frequency. They are also highly range-dependent and hence require a fine knowledge of the water (e.g., temperature, conductivity, speed of sound) and subterrain (e.g., nature, sediments’ density) properties along the path linking a source to a receiver. We discuss here how these site-dependent properties were retrieved for our zone of interest.

**Figure 3:**
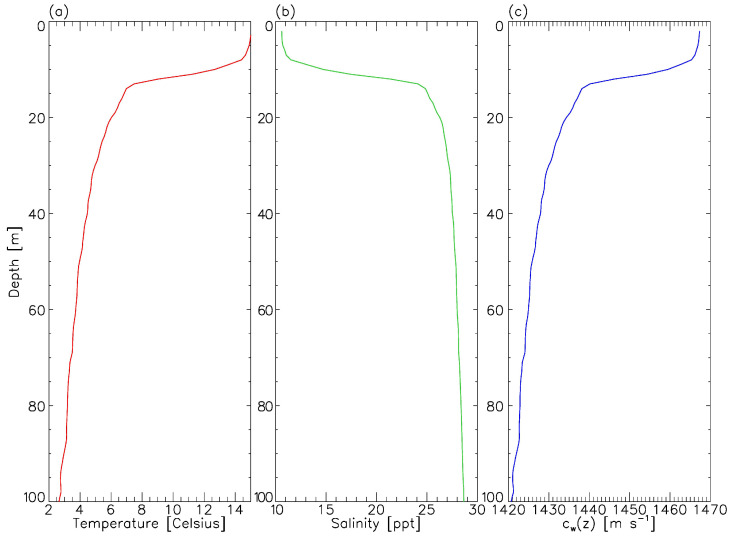
Physicochemical properties of the water column in Anse-Saint-Étienne during summer. Panel (**a**): average water temperature profile. Panel (**b**): average water salinity profile. Panel (**c**): corresponding average speed of sound profile. CTD data were retrieved from the Observatoire global du Saint-Laurent’s archives and were gathered from June to August between 2006 and 2010 in the Saguenay River. The speed of sound profile is dominated by the drop in temperatures measured at depths greater than 10 m.

According to the mooring design, the receiver’s depth, $z_{r}$ , was provided by,

(7) $$z_{r}\;\equiv \;z_{0}\;-\;1.0\;=\;\left\{\begin{array}{cc} 96\;m, & \mathrm{for}\;2021,\\ 92\;m, & \mathrm{for}\;2022. \end{array}\right.$$

where the $z_{0}$ is the height of the water column at the hydrophone’s position (see Section 2.2). With no information regarding the vessels’ draught, the source’s depth, $z_{s}$ , was estimated at 1 m. Bathymetric data of the Saguenay River were retrieved from the Canadian Hydrographic Service and interpolated on a 100-meter mesh grid. The sediments’ nature was obtained from the geological survey of [31], which revealed silt in our zone of interest. According to Table 1.3 of [37], the geoacoustical properties of the seabed were 1575 m s^−1^, 1700 kg m^−3^ and 1.0 dB $\lambda_{p}^{-1}$ , respectively, for the compressional speed of sound ( $c_{b}$ ), density ( $\rho_{b}$ ), and compressional wave attenuation ( $\alpha_{p}$ ) in the subterrain. Average water temperature and salinity profiles (with a 1-m resolution along the depth axis) were provided by the Observatoire global du Saint-Laurent close to our zone of interest (see respectively Figure 3a,b). In Figure 3c, the corresponding speed of sound value at depth *z*, $c_{w}\left(z\right)$ , was provided by [38] (Equation (2)). A high-order polynomial fit was applied to the resulting $c_{w}\left(z\right)$ data and coefficients were stored and later used to construct RAM and Bellhop input files (e.g., see Figure 2 of [39]).

The transmission loss due to magnesium sulfate and boric acid absorbing contributions (TL_abs_) was treated according to the theory developed by [40,41] with a salinity and water acidity of 18‰ (see Figure 3b) and 8, respectively. The water temperature at the time of each recording in Table 1 was provided by the output log file of the hydrophone, ranging between 2.44 °C and 3.72 °C, in agreement with Figure 3a at the depth of deployment (see Equation (7)).

Appendix C discusses how monopole source noise levels (MSLs) were computed from RLs spectra at the CPA. Equation (A3) was looped across the frequency domain to produce MSLs spectra between $f_{0}$ and $f_{1}$ by adding RLs to the total transmission loss (i.e., geometric dilution and absorbing contributions) sustained between the different sources and the receiver’s position. Considering the intermediate depths and the relatively large frequency bandwidth characterizing this work, the RAM model [34] using the paraxial approximation of the Helmholtz equation and the ray-tracing approach [35] were used to estimate the geometric dilution of the sound signal within their respective frequency domain (Table 1 of [36]), and with hydrometric (Observatoire global du Saint-Laurent) and geological [31] input data referenced therein.

Following frequency integration, the RLs and MSLs spectra provided, respectively, the broadband received levels (BB_RL_; see Equations (A1) and (A2)) and broadband monopole source levels (BB_MSL_; see Equations (A5) and (A6)).

### 3.5. Generalized Linear Mixed Model

The minimization of the Akaike information criterion was used to assess the dependency between the vessels’ BB_MSL_ and different observational parameters (see Section 4) during transits at the CPA. A generalized linear mixed model (GLMM) analysis was carried out in R using the function *lmer* of the *lme4* package [42]. The time of the day during CPA occurrences, which should not impact the received noise levels if we choose to neglect absorbing effects caused by microbubbles produced during photosynthesis (see [43]), was used as the random parameter. Confidence intervals and *p*-values (via Wald statistics approximation) were calculated with the function *sjt.lmer* of the *sjPlot* package [44].

## 4. Results

**Figure 4:**
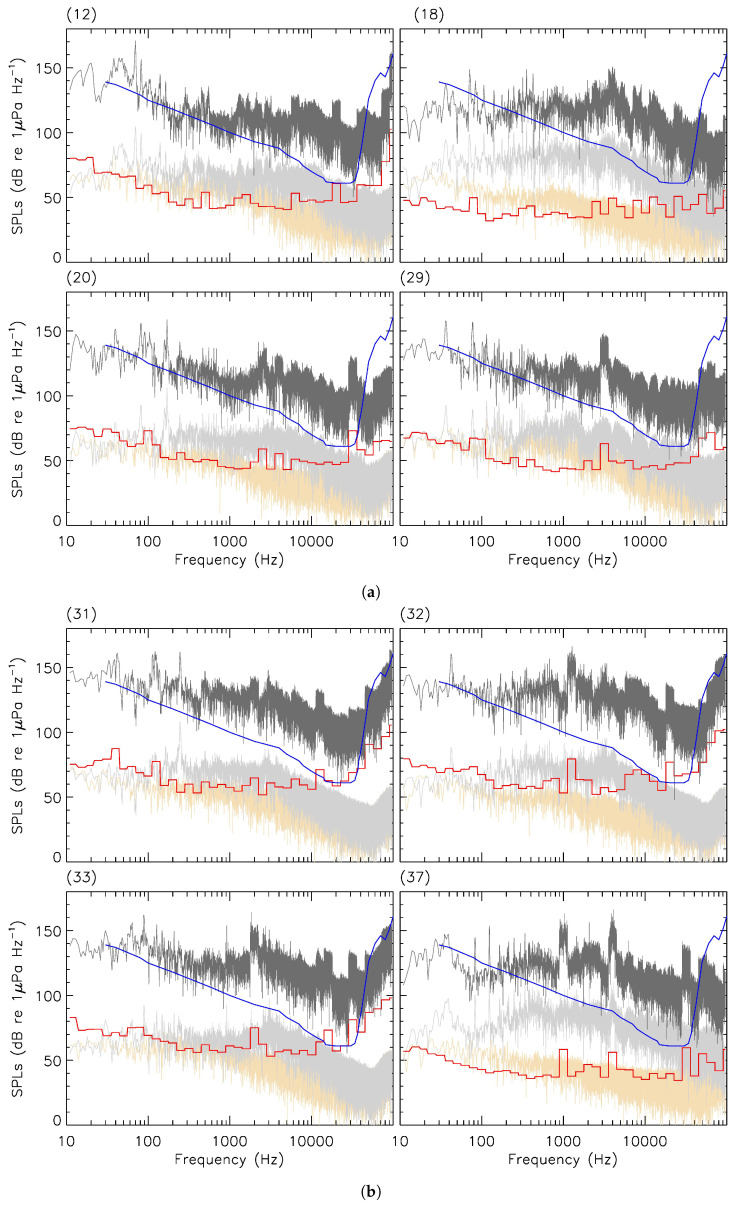

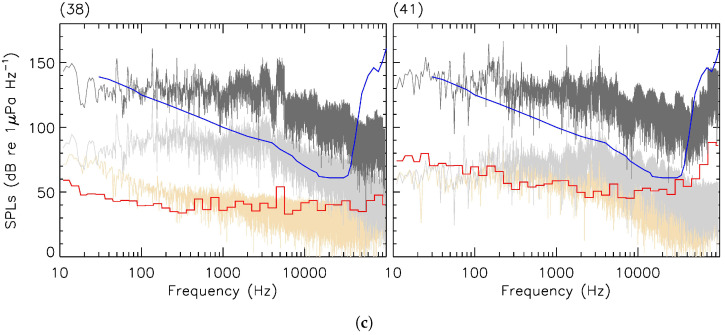
Examples of sound pressure levels (dB re 1 μPa Hz^−1^) in the frequency domain for events of isolated recreational vessel during the 2021–2022 campaigns along the Saguenay River. Light grey: received levels at the hydrophone for CPA occurrences listed in Table 1. Red: transmission loss along the vessel-to-hydrophone transect as predicted by the parabolic equation solver (*f* < *f*_0.5_) and the ray-tracing approach (*f* ≥ *f*_0.5_). Dark grey: monopole source levels of the targeted vessel obtained from the passive SONAR equation (Equation (A3)). Blue: beluga hearing audiogram [45]. Wheat: ambient levels during the 10 min duration (see examples in Figure 2) centred on CPA occurrences. Panels’ numbers refer to the vessels’ labels in Table 1. (**a**) Events 12, 18, 20, and 29; (**b**) events 31, 32, 33, and 37; (**c**) events 38 and 41.

The average CPA distance for the events reported in this work was 403 ± 264(1 σ ) m including five short-range occurrences (≲100 m). TL curves for noteworthy examples of CPA occurrences are shown in red in Figure 4. TL_RAM_ and TL_Bellhop_ were joined at $f_{0.5}$ (see Appendix B).

From Equation (A3), MSLs spectra are shown in dark grey for the few examples of Figure 4. Results for broadband measurements (BB_RL_, BB_MSL_) in both low- and mid-frequency domains are provided in Table 2, where the ambient sound levels (BB_amb._) were estimated, from each corresponding spectrogram, using the time of the lowest signal integrated from $f_{0}$ to $f_{1}$ . This ambient spectrum is shown for each example of Figure 4 as the yellowish wheat curve. The signal excess at the CPA of received levels BB_RL_ with respect to the surrounding ambient noise ranged from 4 (*signal-to-noise*∼2.5) to 41 (*signal-to-noise* > 10,000) dB re 1 μPa. Ambient sound levels at low frequencies (*f* < *f*_0.5_) showed a moderate dispersion with an average of 92.94 ± 5.48(1 σ ) dB re 1 μPa, while the same measurements at mid frequencies (*f* ≥ *f*_0.5_) were fairly constant with an average of 93.88 ± 1.64(1 σ ) dB re 1 μPa.

**Figure 5:**
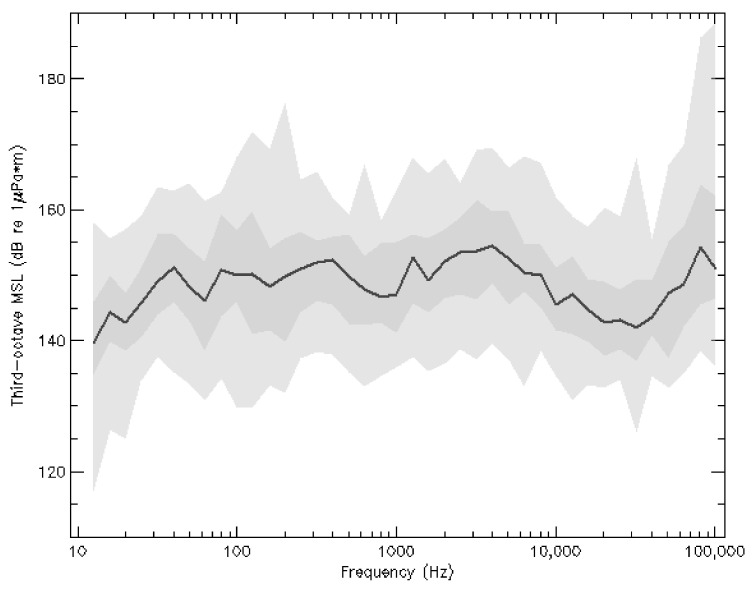
In black, median MSLs spectrum of the 45 events discussed in this work following 1/3-octave band integration. In grey and light grey are shown respectively the 25–75% and 5–95% percentile envelopes.

All 45 MSLs spectra were converted into 1/3-octave bands. Figure 5 shows our sample’s median spectrum along with 25–75% and 5–95% percentile envelopes. The roughly constant 145–150 dB re 1 μPa · m (1/3-octave)^−1^ plateau largely contrasted with the decreasing-with-frequency trend typically found for merchant vessels and the low-frequency emission peaks often above 180 dB re 1 μPa · m (1/3-octave)^−1^, e.g., see Figure 10 of [46].

SOGs, CPA distances, and vessel types in Table 1 were considered as fixed parameters in the GLMM subroutine (see Section 3.5). Results are shown in Table 3a,b. We verified that the GLMM output models were statistically reliable using the R package *DHARMa* version 0.4.5 [47].

**Figure 6:**
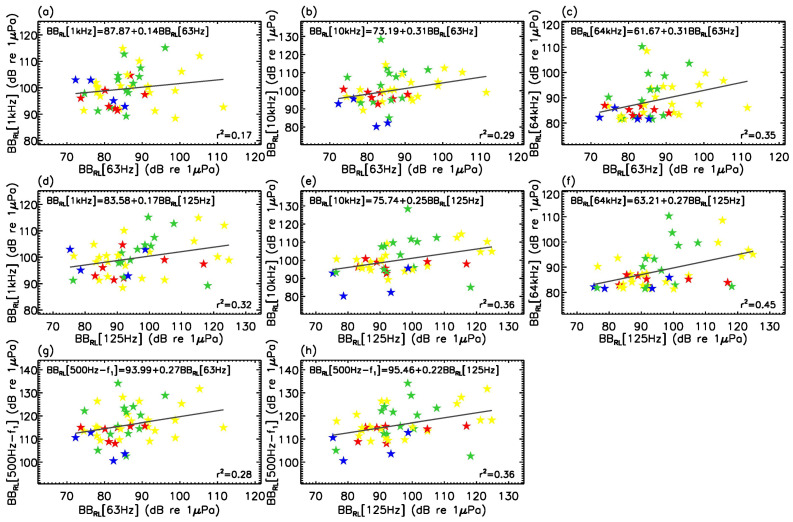
Relationships between received noise levels in the low-frequency 1/3-octave bands, 63 and 125 Hz, and received noise levels in mid-frequency bands for vessels listed in Table 2. BB_RL_ were processed according to the method described in Appendix B with the lower and upper bounds of the summation being provided by each band’s $f_{low}$ and $f_{high}$ values in Table A1. Panels (**a**–**c**): correlations between the 63 Hz band and 1, 10, and 64 kHz bands. Panels (**d**–**f**): correlations between the 125 Hz band and 1, 10, and 64 kHz bands. Panels (**g**,**h**): correlations between the 63 and 125 Hz bands and frequency-integrated broadband between 500 Hz and $f_{1}$ (see Section 3.2). Zodiac^TM^, speedboats, cruisers, and sailboats are respectively coloured in red, yellow, green, and blue. Standard linear regression and corresponding Pearson correlation coefficient are provided, respectively, in the upper-left and bottom-right corners of each panel.

Low-frequency proxies at 63 and 125 Hz, used as approximations of the mid-to-high-frequency noise levels, are explored in Figure 6 in accordance with [48]. Linear fits and statistical correlations are provided in each panel.

## 5. Discussion

Asymmetries in the detectable signal were commonly found in Figure 2 in agreement with [49]. The skewness could be negative (e.g., see events (3), (4), and (10)) for some approaching vessels or positive (e.g., see events (1), (5), and (15)) for others that were distanced from the hydrophone. In light grey for events (12), (20), and (29) of Figure 4, narrow spikes of received signal at low frequencies were likely correlated with the angular frequency of the propellers’ blades ([2], see authors’ Section 5.3). This could not be quantitatively demonstrated, however, due to the lack of information available regarding the propulsion characteristics (i.e., gearbox ratio, RPM, number of blades) of the vessels investigated in this work. Up to about 30 kHz in Figure 4, the propagation at low frequencies (< $f_{0.5}$ ) was typically less efficient in these relatively shallow waters when compared to the propagation at mid frequencies (> $f_{0.5}$ ) i.e., TL_RAM_ > TL_Bellhop_.

**Figure 7:**
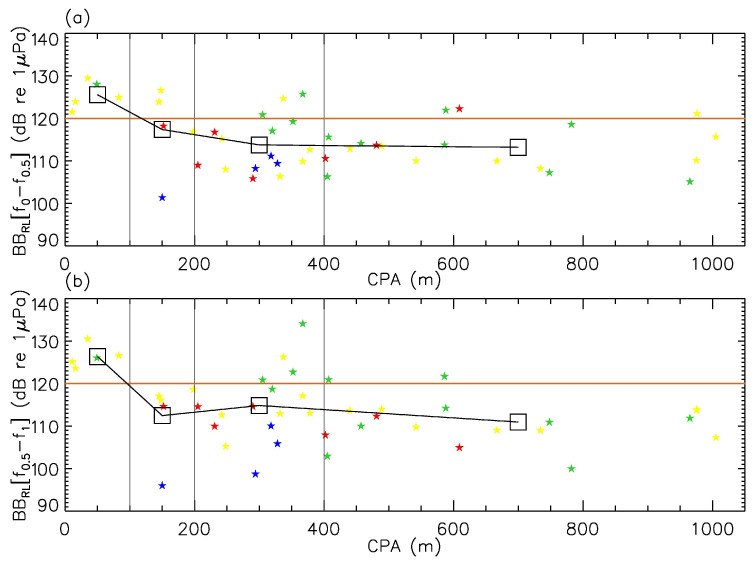
Relationships between CPA distances and broadband received noise levels (**a**): low frequencies, (**b**): mid frequencies. Data were retrieved from Table 1 and Table 2. Zodiac^TM^, speedboats, cruisers, and sailboats are coloured in red, yellow, green, and blue, respectively. Black curves give the average noise level for CPA distances less than 100 m, between 100 and 200 m, between 200 and 400 m, and above 400 m. In both panels, the horizontal brown line indicates the 120 dB re 1 μPa behavioural-disturbance threshold (see text).

The impact of noise pollution on mid-frequency cetaceans is determined by the amplitude of the received signal above $f_{0.5}$ while low-frequency peaks caused by the propellers’ rotation and subsequent harmonics are of less concern [50]. With only a few exceptions (e.g., see events (18), (37), and (38) in Figure 4), RLs spectra above 2–3 kHz typically fell below the beluga hearing audiogram, indicating that these vessels would have caused no specific auditory stresses to a species’ individual located directly at the position of the hydrophone. Figure 7 shows how the received noise levels at the hydrophone decreased with greater CPA distances. Our results tend to support the 400 m minimum distance of approach for boats with respect to the St. Lawrence Estuary beluga suggested by Canada’s Marine Mammals Regulations [51], although variability caused by, e.g., slow/fast SOGs, low/high RPM, or relatively noisy engines, still led to broadband noise levels in excess of the 120 dB re 1 μPa behavioural-disturbance threshold [52] at approach distances greater than 400 m and up to nearly 1 km. Event (41) of Figure 4 reveals received noise levels above the beluga hearing audiogram near 20 kHz at a CPA distance of 586 m (Table 1).

On average, low-frequency source levels BB_MSL_ [ $f_{0}$ – $f_{0.5}$ ] barely exceeded mid-frequency BB_MSL_ [ $f_{0.5}$ – $f_{1}$ ] by 0.92 ± 8.99(1 σ ) dB re 1 μPa · m. Table 3a,b suggested positive correlations between CPA distances and BB_MSL_ in both frequency domains. This indicates that the method used for calculating source levels, no matter how sophisticated, will have some inherent errors. Geometry parameters (e.g., surface grazing angle) are commonly added to regression analyses to remove systematic trends induced by long-range source-to-receiver interactions [14]. The uncertainties on computed source levels were in part attributed to the backpropagated signal that was not emitted at the source but rather inherent to the hydrophone’s direct vicinity. Low frequencies at low tides that were high-pass-filtered by shallow environments and mid-to-high frequencies absorbed before they could reach the receiver in long-range interactions (e.g., see events (31)–(33) in Figure 4) are such examples. Restraining the allowed bandwidth could help minimize these effects. Broadband source levels were again computed after the lower frequency threshold of the low-frequency domain was moved up from $f_{0}$ to 0.1 kHz and, similarly, the higher frequency threshold of the mid-frequency domain was moved down from $f_{1}$ to 30 kHz. Results for both new frequency domains (i.e., 0.1 kHz to $f_{0.5}$ and $f_{0.5}$ to 30 kHz) are shown in columns 5 and 9 of Table 2. The average difference BB_MSL_ [0.1 kHz– $f_{0.5}$ ] – BB_MSL_ [ $f_{0.5}$ –30 kHz] gave 3.13 ± 9.50(1 σ ) dB re 1 μPa · m. Our results hence suggested that the low-frequency mechanical noise of the small sample explored in this work superseded the mid-frequency cavitation by a factor of two in terms of acoustic power. This exposed the beluga’s communication call band (<10 kHz) to potential auditory masking in agreement with the conclusions reached by [53] for similar ship classes and a similar location.

**Figure 8:**
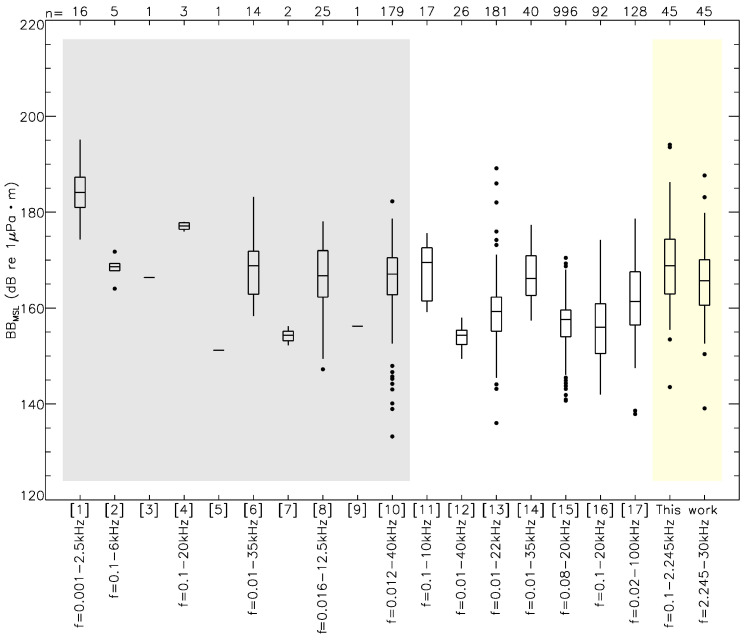
Broadband source levels gathered in [56]’s meta-analysis. Studies shown with the light grey background were not used in the authors’ analysis. References are provided as follows; [1]: [58], [2]: [59], [3]: [60], [4]: [53], [5]: [61], [6]: [54], [7]: [62], [8]: [63], [9]: [64], [10]: [65], [11]: [2], [12]: [5], [13]: [22], [14]: [66], [15]: [23], [16]: [67], and [17]: [55]. The bandwidth applied for each study is provided below each study’s number (that information is missing for studies [3], [5], [7], and [9]). The number of individual measurements (*n*) in each study is provided along the top abscissa. Boxplots are traced out for 5%, 25%, 75%, and 95% percentiles while the middle horizontal line is each sample’s mean. Outliers are shown as black dots. Our contribution to this figure is shown with the light yellow background along both low- (0.1 kHz to $f_{0.5}$ ; see text) and mid-frequency ( $f_{0.5}$ to 30 kHz; see text) domains. From references [1] to [17], the figure was reproduced with permission from Miles J. G. Parsons, A Review and Meta-Analysis of Underwater Noise Radiated by Small (<25 m Length) Vessels; published by Journal of Marine Science and Engineering, 2021.

The values for BB_MSL_ [0.1 kHz– $f_{0.5}$ ] and BB_MSL_ [ $f_{0.5}$ –30 kHz] listed in Table 2 were coherent with other studies of similar vessel classes (e.g., [54,55]). Figure 8 was reproduced from [56] in which we added our contribution to the authors’ work. In a similar but parameter-controlled experience carried out at the mouth of the Saguenay River, (Figure 13 of [57]) found similar source levels in 1/3-octave bands for small recreational vessels (<10 m) as those shown in this work’s Figure 5 although the ∼6 dB re 1 μPa · m octave^−1^ monotonic decrease from 100 Hz to 25 kHz measured by the authors could not be retrieved here.

In Table 3c,d, the correlation between source levels and SOG was positive, ranging from 0.40 to 0.68 dB knots^−1^. This result agreed with the generally accepted trend that source levels likely increase with increasing speed (see, e.g., [11,68,69,70]) even though these studies mostly focused on large military or merchant ships and other vessel classes that were not necessarily representative of the sound radiating by recreational vessels. Ref. [57], for vessel classes similar to ours, obtained a 0.5–1 dB knots^−1^ correlation, giving credence to our findings.

No specific correlation could be established between source levels reported in this work and the four types of vessels explored in this study’s sample. The geometric bias of the CPA parameter was statistically attenuated from Table 3b to Table 3d, as seen by its *p*-value increasing from <0.001 to 0.064. This suggests that, for a majority of the 45 events discussed in this work, most (if not all) of the noise levels measured at frequencies above 30 kHz could correspond to very localized noise emitted at or near the hydrophone.

Better correlations ( $r^{2}$ ≳ 0.3) between low-frequency proxies and mid-frequency bands were found in Figure 6 when compared to [50] (Figure 4). As opposed to the GLMM approach discussed above, substituting $f_{1}$ for 30 kHz in Figure 6g,h yielded no statistically relevant variations on the linear regressions and correlation coefficients calculated.

## 6. Conclusions

This work provided the received noise levels, transmission loss calculations, and monopole source levels of isolated motorized recreational vessels through opportunistic hydrophone-based recordings. Many of the recommendations suggested by [56] were followed in this study, in particular the consideration of the low-frequency energy ( $f_{0}$ < 50 Hz) and robust numerical backpropagation.

The main results of our study are:

1. The received noise levels associated with the passage of one small recreational vessel at a time raised the ambient levels in the given integrated frequency bands by 4 to 41 dB re 1 μPa. The average signal excess was about 22 dB re 1 μPa in both frequency domains explored.
2. At least 31.1% of the recorded targets (14 out of 45 events) in Anse-Saint-Étienne have shown received noise levels in excess of the St. Lawrence Estuary beluga hearing audiogram, hence suggesting evidence for acoustic disturbance at CPA distances of a few hundreds meters. In those specific cases, both the beluga’s communication and echolocation bands have increased risks of auditory masking during short-to-intermediate range interactions (<600 m) (see Appendix D).
3. Across restrained bandwidths between 0.1 and 30 kHz, monopole source levels were, on average, 3 dB re 1 μPa · m higher at low frequencies when compared to their mid-frequency counterparts. This revealed the importance of the low-frequency domain in the investigation of such vessels.
4. Across restrained bandwidths between 0.1 and 30 kHz, a positive correlation between computed source levels and the speed over ground at low and mid frequencies agreed with the commonly accepted proposition that increasing speeds is usually translated into increasing radiated noise levels.
5. The geometric bias towards large CPAs (∼1 km) could be related to a high-frequency signal (>30 kHz) either emitted by a very nearby source or simply part of the ambient noise and erroneously treated as being emitted by the passing vessel. The backpropagation of such signal yielded unrealistically large source levels that kept increasing with the CPA distance.
6. Moderate correlations between the low- and mid-frequency received noise levels were obtained. Low-frequency proxies seemed to be modest predictors of the acoustic power radiated by small vessels at mid-to-high frequencies.

## Figures and Tables

**Table 1:** Shore-based Observations of Isolated Small Recreational Vessels. Col. 1: vessel’s ID; same as trajectories in Figure 1. Col. 2: date of the observation. Col. 3: local time of the observation. Col. 4: local sea state. Col. 5: type of vessel. Cols. 6 to 8: number, manufacturer, and total power of engines. Col. 9: vessel’s speed over ground. Cols. 10 and 11: latitude and longitude of the vessel at the CPA. Col. 12: vessel-to-hydrophone distance at the CPA.

Label	Date	Time (EDT)	Beaufort Sea State	Type	Number of Engines	Engines Manufacturer	Engines Power (HP)	SOG (knots)	$\phi_{i,\mathrm{CPA}}$ (°)	$\lambda_{i,\mathrm{CPA}}$ (°)	CPA (m)
1	31 July 2021	13:55	2	Speedboat	-	-	-	1.88	48.213861	−69.887152	667
2	3 August 2021	16:01	1	Speedboat	1	Mercury	-	23.08	48.210289	−69.896352	367
3	4 August 2021	15:50	0	Cruiser	-	-	-	30.17	48.208510	−69.892474	49
4	5 August 2021	10:32	0	Cruiser	-	-	-	8.87	48.210237	−69.886181	457
5 *	5 August 2021	12:08	0	Zodiac^TM^	1	Yamaha	150	10.02	48.211068	−69.899422	609
6 *	5 August 2021	12:33	0	Zodiac^TM^	1	Suzuki	-	20.10	48.210073	−69.888555	290
7	7 August 2021	09:06	1	Speedboat	-	-	-	28.39	48.208786	−69.892162	16
8	7 August 2021	11:30	1	Speedboat	1	Mercury	115	21.04	48.208475	−69.893851	145
9	7 August 2021	12:50	1	Cruiser	-	-	-	17.47	48.209724	−69.881958	748
10	10 August 2021	13:28	1	Cruiser	-	-	-	17.56	48.209916	−69.888201	305
11	12 August 2021	16:20	1	Speedboat	-	-	-	17.59	48.209490	−69.897792	440
12	13 August 2021	14:29	0	Speedboat	2	Mercury	115	6.79	48.207001	−69.898766	542
13	16 August 2021	10:35	1	Cruiser	-	-	-	20.35	48.209907	−69.887981	320
14	18 August 2021	12:56	1	Speedboat	1	Mercury	-	20.97	48.209603	−69.888929	242
15	25 August 2021	15:54	1	Speedboat	-	-	-	21.05	48.207462	−69.889247	248
16	1 September 2021	11:54	2	Speedboat	1	Mercury	115	18.95	48.208877	−69.893944	148
17	15 July 2022	14:16	2	Cruiser	-	-	-	9.03	48.212062	−69.887515	782
18	16 July 2022	11:08	2	Speedboat	1	-	-	24.49	48.209266	−69.893283	11
19	16 July 2022	13:03	2	Speedboat	-	-	-	21.51	48.207783	−69.889580	332
20	16 July 2022	13:45	2	Zodiac^TM^	1	-	-	16.28	48.211091	−69.888693	402
21	16 July 2022	15:05	2	Speedboat	-	-	-	11.12	48.207778	−69.899595	489
22	18 July 2022	09:35	1	Sailboat	-	-	-	7.74	48.209975	−69.889102	328
23	18 July 2022	10:29	1	Zodiac^TM^	1	-	115	17.97	48.210392	−69.892154	152
24 *	18 July 2022	11:17	1	Speedboat	1	-	-	22.22	48.210675	−69.889347	337
25	18 July 2022	11:43	1	Zodiac^TM^	1	Yamaha	150	7.13	48.208487	−69.896273	231
26	25 July 2022	15:54	2	Speedboat	1	-	-	22.31	48.210086	−69.880296	976
27	126 July 2022	10:31	2	Zodiac^TM^	2	-	-	16.25	48.210202	−69.890991	205
28	26 July 2022	13:44	2	Zodiac^TM^	2	-	-	17.10	48.210971	−69.887420	481
29	28 July 2022	09:58	2	Sailboat	-	-	-	6.62	48.210829	−69.889774	318
30	28 July 2022	12:24	2	Cruiser	-	-	-	21.32	48.211299	−69.886062	588
31	28 July 2022	13:04	3	Speedboat	1	-	-	22.58	48.211086	−69.880111	1005
32	30 July 2022	09:08	1	Speedboat	1	-	-	29.52	48.209972	−69.880295	975
33	30 July 2022	10:03	1	Cruiser	1	-	-	17.16	48.210751	−69.880569	965
34	30 July 2022	13:54	2	Speedboat	-	-	-	18.99	48.208905	−69.896018	198
35	31 July 2022	08:55	2	Sailboat	-	-	-	8.50	48.210479	−69.889857	294
36	31 July 2022	11:35	2	Cruiser	-	-	-	23.50	48.210530	−69.888811	367
37	31 July 2022	12:28	2	Speedboat	1	-	-	25.56	48.209704	−69.892451	83
38	31 July 2022	15:09	2	Speedboat	-	-	-	20.52	48.209070	−69.893708	35
39	1 August 2022	16:42	2	Sailboat	-	-	-	6.51	48.209702	−69.891473	150
40	2 August 2022	14:20	2	Cruiser	-	-	-	22.58	48.210806	−69.888402	407
41	3 August 2022	09:34	2	Cruiser	-	-	-	20.75	48.212184	−69.886782	586
42	13 August 2022	11:07	2	Cruiser	-	-	-	5.67	48.211005	−69.888579	405
43	15 August 2022	15:37	2	Cruiser	-	-	-	25.03	48.208605	−69.898050	352
44	17 August 2022	09:49	2	Speedboat	-	-	-	24.34	48.211014	−69.883850	734
45	17 August 2022	10:11	2	Speedboat	2	-	115	10.08	48.207896	−69.898055	378

* Presence of belugas noted.

**Table 2:** Broadband Measurements of Isolated Small Recreational Vessels. Col. 1: vessel’s ID; same as trajectories in Figure 1. Cols. 2 to 5: ambient, received, full-band source, and restrained band source levels in the low-frequency domain. Cols. 6 to 9: ambient, received, full-band source, and restrained band source levels in the mid-frequency domain.

Label	Low Frequencies				Mid Frequencies			
	BB_amb._ [ $f_{0}$ – $f_{0.5}$ ] (dB re 1 μPa)	BB_RL_ [ $f_{0}$ – $f_{0.5}$ ] (dB re 1 μPa)	BB_MSL_ [ $f_{0}$ – $f_{0.5}$ ] (dB re 1 μPa · m)	BB_MSL_ [0.1 kHz– $f_{0.5}$ ] (dB re 1 μPa · m)	BB_amb._ [ $f_{0.5}$ – $f_{1}$ ] (dB re 1 μPa) (dB re 1 μPa)	BB_RL_ [ $f_{0.5}$ – $f_{1}$ ] (dB re 1 μPa · m)	BB_MSL_ [ $f_{0.5}$ – $f_{1}$ ]	BB_MSL_ [ $f_{0.5}$ –30 kHz] (dB re 1 μPa · m)
1	96.16	110.01	186.72	169.37	95.38	109.05	174.72	168.60
2	87.51	109.91	161.17	160.61	93.22	117.10	168.99	168.43
3	93.76	128.02	170.25	169.94	94.27	126.03	166.72	166.62
4	97.89	114.13	176.77	174.88	95.62	109.99	166.55	163.25
5	97.12	122.30	186.49	186.23	95.58	104.94	169.83	155.58
6	97.51	105.85	161.35	155.76	95.45	114.63	169.02	167.20
7	94.45	123.93	164.61	164.55	95.64	123.59	168.00	167.88
8	98.22	123.89	170.08	169.45	95.78	117.02	167.04	164.88
9	94.28	107.26	176.10	175.77	93.78	110.90	184.03	183.12
10	96.80	120.88	175.24	168.52	95.16	120.87	171.72	170.08
11	99.68	112.90	168.57	164.67	94.44	113.62	165.20	161.61
12	96.37	110.05	173.44	155.43	93.65	109.76	188.99	158.45
13	100.00	117.08	170.06	169.25	97.77	118.68	171.50	168.87
14	88.64	115.21	174.63	174.35	92.94	112.63	164.15	163.70
15	94.99	108.07	160.52	156.01	93.00	105.25	163.13	162.49
16	97.85	126.59	172.53	172.07	93.98	116.10	161.94	160.20
17	93.66	118.60	185.57	185.44	93.89	99.97	169.86	152.63
18	85.93	121.53	159.78	159.08	92.29	125.12	171.17	171.13
19	93.48	106.33	160.48	153.46	92.61	112.92	161.61	157.30
20	86.81	110.58	165.63	162.91	92.21	107.88	163.89	160.58
21	84.43	113.50	179.95	164.10	92.61	113.97	166.00	163.03
22	90.88	109.42	161.06	160.45	93.03	105.86	159.24	156.61
23	82.36	118.20	168.02	167.37	92.18	114.60	164.27	164.24
24	97.56	124.67	176.85	176.77	94.13	126.26	179.88	179.82
25	102.03	116.77	170.10	169.96	94.71	109.96	158.70	157.33
26	89.28	121.13	179.60	179.56	92.54	113.84	191.64	173.30
27	94.39	108.99	157.50	155.47	94.31	114.58	162.05	161.41
28	95.00	113.70	174.57	172.59	93.61	112.33	170.45	167.71
29	100.77	111.16	164.60	157.52	94.08	110.04	168.42	167.84
30	97.70	121.95	193.55	193.54	94.05	114.21	176.70	171.04
31	90.18	115.68	174.41	173.49	93.14	107.30	192.91	166.69
32	84.64	110.15	180.00	179.89	92.35	113.75	192.86	173.77
33	92.99	105.14	175.75	175.00	92.76	111.85	188.64	170.32
34	93.92	116.89	165.85	165.29	93.98	118.61	159.37	158.58
35	84.41	108.25	165.32	159.06	92.22	98.71	152.98	150.40
36	96.12	125.73	194.10	194.07	93.40	134.10	187.94	187.65
37	83.54	124.93	174.15	174.09	92.27	126.59	180.15	179.85
38	89.60	129.47	169.49	169.10	92.35	130.49	175.57	175.55
39	82.76	101.39	152.26	143.53	92.29	96.00	147.64	139.08
40	101.05	115.64	171.79	168.30	98.38	120.92	174.88	167.85
41	99.55	113.78	174.46	174.12	98.93	121.69	180.51	171.37
42	91.04	106.30	172.03	171.96	92.97	102.91	169.27	168.98
43	86.74	119.29	177.73	164.25	92.44	122.69	169.30	168.58
44	88.80	108.22	176.29	176.19	92.62	108.98	175.42	163.17
45	91.40	112.68	171.60	163.92	92.51	113.05	166.80	159.67

**Table 3:** Generalized Linear Mixed Models.

Predictor	Estimate	Confidence Interval	*p*-Value
**(a) BB_MSL_ [** $f_{0}$ **–** $f_{0.5}$ **] (dB re 1 μPa ·m)**			
Intercept	161.60	153.26–169.95	**<0.001**
SOG	0.11	−0.22–0.44	0.515
CPA	0.02	0.01–0.03	**<0.001**
Type	0.04	−2.75–2.82	0.980
**(b) BB_MSL_ [** $f_{0.5}$ **–** $f_{1}$ **] (dB re 1 μPa · m)**			
Intercept	149.70	142.10–157.31	**<0.001**
SOG	0.60	0.31–0.90	**<0.001**
CPA	0.03	0.02–0.03	**<0.001**
Type	−0.05	−2.49–2.38	0.966
**(c) BB_MSL_ [0.1 kHz–** $f_{0.5}$ **] (dB re 1 μPa · m)**			
Intercept	153.77	143.87–163.66	**<0.001**
SOG	0.40	0.02–0.78	**0.040**
CPA	0.02	0.01–0.03	**<0.001**
Type	0.05	−3.14–3.25	0.974
**(d) BB_MSL_ [** $f_{0.5}$ **–30 kHz] (dB re 1 μPa · m)**			
Intercept	150.34	142.16–158.52	**<0.001**
SOG	0.68	0.36–0.99	**<0.001**
CPA	0.01	−0.00–0.02	0.064
Type	0.47	−2.20–3.15	0.729

**Figure A1:**
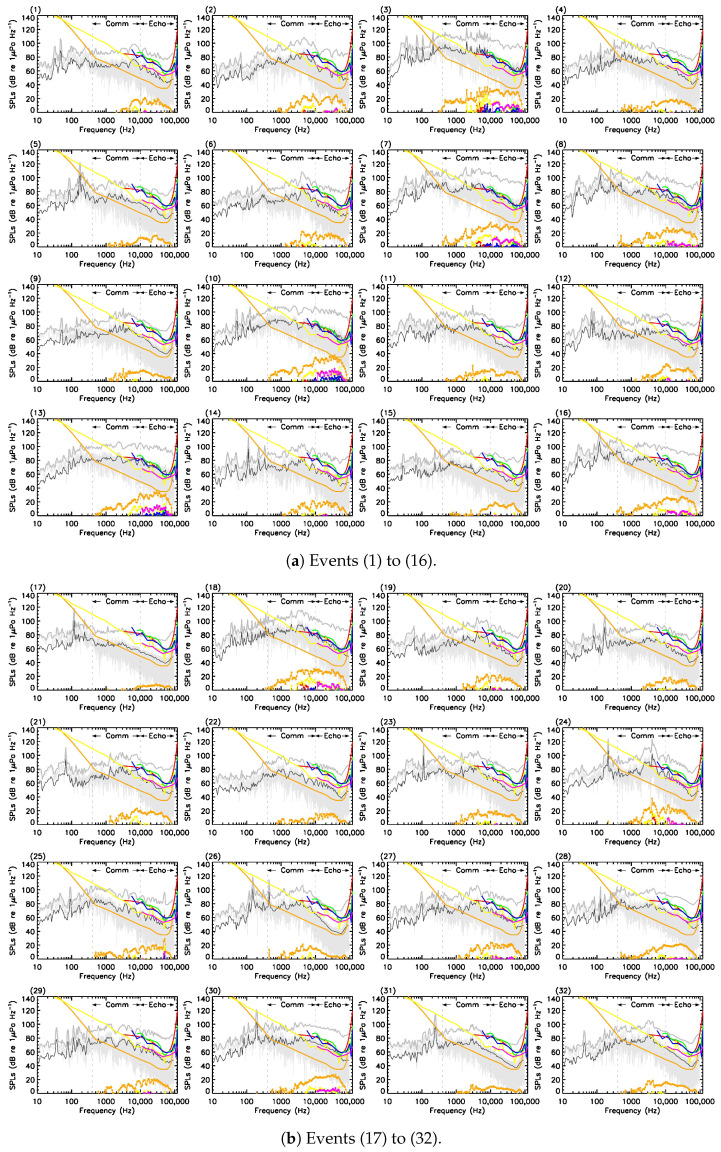

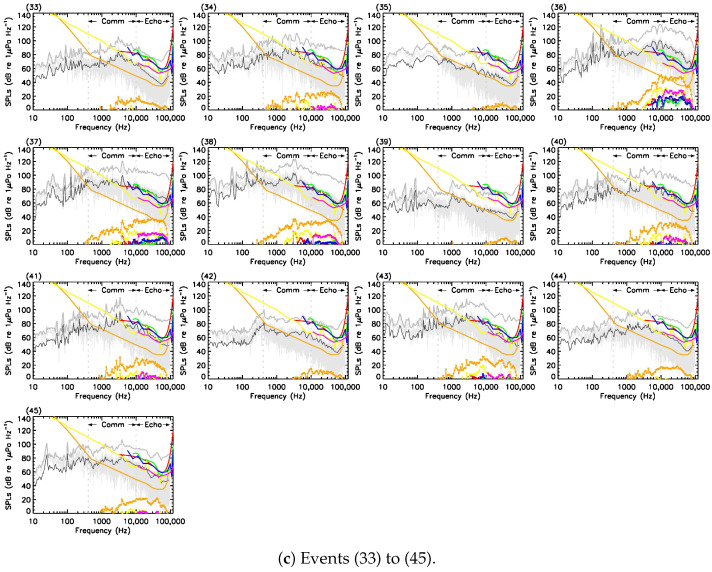
Acoustic disturbance in Anse-Saint-Étienne. Each panel (and label) corresponds to a specific event in this work’s Table 1. At CPA, RL levels (in units of dB Hz^−1^) recorded at the hydrophone are shown in light gray. The same signal expressed in twelfth-octave bands (in units of dB) is provided in gray. Subtraction of the frequency-dependent critical ratios [75] from the twelfth-octave bands yields the black curve. Six (6) audiograms for the beluga whale are provided: red [77], orange [5], yellow [45], green [78], blue [79], and magenta [80]. Subtraction of each audiogram from the black curve gives the color-corresponding circle-chained line (referred to as excesses in the text) in the lower portion of each panel. Beluga’s communication and echolocation bands are represented by the vertical dashed gray lines.

## Data Availability

Not applicable.

## Abbreviations

The following abbreviations are used in this manuscript:

AIS	Automatic Identification System
BB	Broadband
CPA	Closest point of approach
EDT	Eastern Daylight Time
GLMM	Generalized linear mixed model
GREMM	Groupe de Recherche et d’Éducation sur les Mammifères Marins
MSL	Monopole source level
RL	Received level
SOG	Speed over ground
SPL	Sound pressure level
TL	Transmission loss

## Appendix A. Data Processing Following Hydrophone Retrieval

Local times in this work were given according to the Eastern Daylight Time (EDT) zone (i.e., UTC-04:00). WAV files of the acoustic soundscape were recorded in continuous segments of 90 min, eight times a day starting at the top of the hour, between 08:00:00 EDT and 19:59:59 EDT. Matlab^®^-supported PAMGuide [30] was used to convert the recorded WAV files into the frequency domain through a power spectral density analysis by estimating the mean sound pressure levels (SPLs) in 1-second time windows. SPLs (in dB re 1 μPa Hz^−1^) were extracted using the Welch method [71] with a Hann window [72] and 50% temporal overlap. SPLs are hereafter referred to as received noise levels (RLs) at the hydrophone’s position. Hence, 43200 RL spectra between $f_{0}$ and $f_{1}$ (see Section 3.2) were processed each day, one for each second during the 12-hour period when the hydrophone was enabled for recording.

## Appendix B. Broadband RLs Computation

One-third-octave bands between $f_{0}$ and $f_{1}$ are listed in Table A1. We defined $f_{0.5}$ ≡ 2.245 kHz as the limit between low and mid frequencies in this work (see details in Appendix C). All 45 RLs spectra corresponding to CPA occurrences (see examples of these spectra in light grey in Figure 4) were spectrally collapsed, first between $f_{0}$ and $f_{0.5}$ and then between $f_{0.5}$ and $f_{1}$ , to obtain broadband measurements (in dB re 1 μPa), respectively, in the low-(BB_RL_ [ $f_{0}$ – $f_{0.5}$ ]) and mid-frequency (BB_RL_ [ $f_{0.5}$ – $f_{1}$ ]) domain of the acoustic signal received at the hydrophone. Hence,

(A1)
$${\mathrm{BB}}_{\mathrm{RL}}\left[f_{0}–f_{0.5}\right]=10.0\;\times \;\log_{10}\left(\sum_{f=f_{0}}^{f_{0.5}}10^{\mathrm{RL}(f)/10}\right),$$

(A2)
$${\mathrm{BB}}_{\mathrm{RL}}\left[f_{0.5}–f_{1}\right]=10.0\;\times \;\log_{10}\left(\sum_{f=f_{0.5}}^{f_{1}}10^{\mathrm{RL}(f)/10}\right).$$

**Table A1:** 1/3-Octave Bands.

$f_{\mathrm{low}}$	$f_{c}$	$f_{\mathrm{high}}$
**(Hz)**	**(Hz)**	**(Hz)**
11	12	13
14	16	17
18	20	21
22	25	27
28	32	35
36	40	44
45	50	55
56	63	70
71	80	88
89	100	111
112	125	140
141	160	177
178	200	223
224	250	281
282	315	354
355	400	446
447	500	561
562	630	707
708	800	890
891	1000	1122
1123	1260	1413
1414	1587	1781
1782	2000	2244
2245	2520	2827
2828	3175	3563
3564	4000	4489
4490	5040	5655
5656	6350	7126
7127	8000	8978
8979	10,080	11,312
11,313	12,700	14,253
14,254	16,000	17,958
17,959	20,160	22,626
22,627	25,400	28,507
28,508	32,000	35,917
35,918	40,320	45,253
45,254	50,800	57,016
57,017	64,000	71,836
71,837	80,640	90,508
90,509	101,600	114,035

## Appendix C. Broadband MSLs Computation

TL_RAM_ and TL_Bellhop_ were processed for each $f_{c}$ values in Table A1 and were assumed constant for each integer frequency inside each band between $f_{low}$ and $f_{high}$ . The definition for $f_{0.5}$ in Appendix B was determined as an approximation (i.e., closest $f_{low}$ value) of the best numerical agreement between the TL_RAM_ and TL_Bellhop_ predictions in the frequency range between 0.5 and 10 kHz.

Frequency-dependent monopole source-level (MSLs) spectra were processed using the passive SONAR equation,

(A3)
$$\mathrm{MSL}\left(f;\phi ,\lambda \right)\;=\;\mathrm{RL}\left(f;\phi_{0},\lambda_{0}\right)\;+\;{\mathrm{TL}}_{X}\left(f;\phi ,\lambda \to \phi_{0},\lambda_{0}\right)\;+\;{\mathrm{TL}}_{\mathrm{abs}}\left(f;\phi ,\lambda \to \phi_{0},\lambda_{0}\right),$$

where TL_abs_(*f*; ϕ , λ → $\phi_{0}$ , $\lambda_{0}$ ) is the sound absorption sustained between the vessels’ position ( ϕ , λ ) and the hydrophone’s position ( $\phi_{0}$ , $\lambda_{0}$ ) at frequency *f* (see Section 3.4), and RL(*f*; $\phi_{0}$ , $\lambda_{0}$ ) and MSL(*f*; ϕ , λ ) are, respectively, the sound levels measured at the hydrophone and the monopole source levels computed at the vessels’ position for frequency *f*. The three parameters were connected by geometric sound dilution using:

(A4)
$${\mathrm{TL}}_{X}\left(f;\phi ,\lambda \;\to \;\phi_{0},\lambda_{0}\right)\;=\;\left\{\begin{array}{cc} {\mathrm{TL}}_{\mathrm{RAM}}\left(f;\phi ,\lambda \;\to \;\phi_{0},\lambda_{0}\right), & \mathrm{if}\;f\;<\;f_{0.5}\\ {\mathrm{TL}}_{\mathrm{Bellhop}}\left(f;\phi ,\lambda \;\to \;\phi_{0},\lambda_{0}\right), & \mathrm{if}\;f\;\geq \;f_{0.5}. \end{array}\right.$$

The spectral collapse of the MSLs spectra obtained from Equation (A4), first between $f_{0}$ and $f_{0.5}$ and then between $f_{0.5}$ and $f_{1}$ , gave broadband measurements (in dB re 1 μPa · m), respectively, in the low-(BB_MSL_ [ $f_{0}$ – $f_{0.5}$ ]) and mid-frequency (BB_MSL_ [ $f_{0.5}$ – $f_{1}$ ]) domain of the acoustic signal radiated at each source. Hence,

(A5)
$${\mathrm{BB}}_{\mathrm{MSL}}\left[f_{0}–f_{0.5}\right]=10.0\;\times \;\log_{10}\left(\sum_{f=f_{0}}^{f_{0.5}}10^{\mathrm{MSL}(f)/10}\right),$$

(A6)
$${\mathrm{BB}}_{\mathrm{MSL}}\left[f_{0.5}–f_{1}\right]=10.0\;\times \;\log_{10}\left(\sum_{f=f_{0.5}}^{f_{1}}10^{\mathrm{MSL}(f)/10}\right).$$

## Appendix D. Proof

To address any form of misinterpretation, we closely followed the method described by Gervaise and collaborators [53, *§* IV-B]. Figure A1 shows, in light gray, the RLs recorded by the hydrophone (in units of dB Hz^−1^) during CPA passage for all 45 events listed in this work’s Table 1. Critical bands of twelfth-octave are generally suited to the beluga’s hearing sensitivity [73,74]. In the frequency domain, the subtraction of critical ratios [75] from the RL twelfth-octave bands leads to a spectrum that can now be directly compared to published hearing audiograms. Positive differences (referred to as excesses), shown in Figure A1 as circle-chained lines, correspond the amount (in dB) of a signal at a given frequency that must be emitted above the audiogram to be detected.

The audiograms in Figure A1 are a mixture of auditory evoked potentials and behavioural hearing thresholds. The main objective of this work was to contribute/add to the sample of known MSLs for motorised recreational crafts (see, e.g., [56]); it is beyond its scope to determine which audiogram is better representative of the beluga’s hearing thresholds and why. In Table A2, masking was judged probable if at least four out six audiograms suggest so with a maximal signal excess required for detection above 4.8 dB (highlighted in coral), i.e., the nominal uncertainty on a single acoustic measurement [76]. All in all, 14 out of 45 events, i.e., 31.1% of our sample in Anse-Saint-Étienne, support evidence for masking at the position of the hydrophone, in addition to 5 contested events.

**Table A2:** Maximal signal excess required for detection and corresponding frequency.

Event	Castellote et al. [77]		Erbe et al. [5]		Finneran et al. [45]		Klishin et al. [78]		Mooney et al. [79]		Sysueva et al. [80]		Masking?
	**Frequency**	**Excess**	**Frequency**	**Excess**	**Frequency**	**Excess**	**Frequency**	**Excess**	**Frequency**	**Excess**	**Frequency**	**Excess**	
	**(kHz)**	**(dB)**	**(kHz)**	**(dB)**	**(kHz)**	**(dB)**	**(kHz)**	**(dB)**	**(kHz)**	**(dB)**	**(kHz)**	**(dB)**	
1	−	−	13.6	21	7.2	8	−	−	−	−	13.6	2	✗
2	4.5	3	8.5	27	8.5	12	−	−	−	−	40.6	6	?
3	4.8	14	8.1	37	6.8	26	57.5	7	8.1	12	15.2	15	✓
4	−	−	20.3	19	7.2	4	−	−	−	−	−	−	✗
5	−	−	28.7	16	−	−	−	−	−	−	−	−	✗
6	4.0	3	4.0	22	6.8	7	−	−	−	−	−	−	✗
7	4.5	8	10.2	35	7.2	17	45.6	6	15.2	5	15.2	14	✓
8	3.0	2	10.8	28	51.2	11	51.2	1	−	−	11.4	8	?
9	−	−	3.2	18	6.8	3	−	−	−	−	−	−	✗
10	36.2	8	36.2	38	51.2	20	54.2	10	36.2	8	36.2	18	✓
11	3.0	5	3.0	23	6.8	6	−	−	−	−	−	−	?
12	−	−	10.8	25	8.5	7	−	−	−	−	12.1	4	✗
13	54.2	7	30.4	36	51.2	18	54.2	11	54.2	7	30.4	15	✓
14	−	−	8.0	21	8.0	8	−	−	−	−	21.5	1	✗
15	−	−	13.6	19	48.3	4	−	−	−	−	−	−	✗
16	−	−	30.4	28	51.2	11	51.2	1	−	−	11.4	7	?
17	−	−	28.7	9	−	−	−	−	−	−	−	−	✗
18	4.0	10	9.1	31	7.2	17	45.6	3	7.6	3	12.8	11	✓
19	−	−	7.6	23	7.6	12	−	−	−	−	19.2	2	✗
20	−	−	13.6	18	9.1	3	−	−	−	−	−	−	✗
21	−	−	8.1	23	7.6	12	−	−	−	−	−	−	✗
22	−	−	10.2	16	−	−	−	−	−	−	−	−	✗
23	4.3	1	4.3	21	7.2	5	−	−	−	−	15.2	1	✗
24	4.0	18	4.0	38	4.0	21	−	−	−	−	12.1	9	✓
25	51.2	4	51.2	29	51.2	18	51.2	8	51.2	4	51.2	10	✓
26	4.3	1	4.2	21	7.2	9	−	−	−	−	−	−	✗
27	4.0	1	10.8	24	7.6	8	−	−	−	−	11.4	4	✗
28	−	−	27.1	22	7.2	6	−	−	−	−	27.1	1	✗
29	−	−	17.1	21	8.1	4	−	−	−	−	17.1	2	✗
30	−	−	38.4	27	48.3	9	48.3	1	−	−	38.4	8	?
31	−	−	3.0	12	−	−	−	−	−	−	−	−	✗
32	−	−	4.3	18	6.8	2	−	−	−	−	−	−	✗
33	3.0	1	12.1	20	7.2	7	−	−	−	−	12.1	0	✗
34	3.8	6	38.4	27	7.2	14	48.3	1	−	−	38.4	8	✓
35	−	−	30.4	10	−	−	−	−	−	−	−	−	✗
36	38.4	19	10.2	50	9.1	33	43.1	20	9.1	20	13.6	29	✓
37	4.0	15	30.4	38	51.2	23	54.2	13	57.5	10	38.4	17	✓
38	3.0	18	3.4	37	7.6	20	51.2	6	8.1	8	13.5	14	✓
39	−	−	38.4	9	−	−	−	−	−	−	−	−	✗
40	3.2	8	18.1	31	9.1	15	51.2	5	18.1	4	18.1	13	✓
41	3.4	13	3.4	31	3.4	13	−	−	16.1	1	15.2	10	✓
42	−	−	10.2	15	−	−	−	−	−	−	−	−	✗
43	3.4	6	8.1	32	8.1	19	48.3	3	8.1	7	11.4	8	✓
44	−	−	30.4	18	7.2	3	−	−	−	−	−	−	✗
45	3.8	1	40.6	22	7.2	6	−	−	−	−	15.2	3	✗
